# The utility of PAX8 and SATB2 immunohistochemical stains in distinguishing ovarian mucinous neoplasms from colonic and appendiceal mucinous neoplasm

**DOI:** 10.1186/s13104-019-4816-9

**Published:** 2019-11-26

**Authors:** Najla Aldaoud, Madiha Erashdi, Sohaib AlKhatib, Nour Abdo, Alia Al-Mohtaseb, Ashley Graboski-Bauer

**Affiliations:** 1 Department of Pathology and Laboratory Medicine, Jordan University of Science and Technology, PO Box 3030, Irbid, 22110 Jordan; 2 Department of Public Health and Community Medicine, Jordan University of Science and Technology, Irbid, Jordan; 3 Department of Public Health Sciences, New Mexico State University, Las Cruces, USA

**Keywords:** Ovary, Mucinous tumor, PAX8, SATB2, Immunohistochemistry

## Abstract

**Objectives:**

It is challenging to distinguish between primary ovarian mucinous tumors and metastatic mucinous neoplasms from the lower gastrointestinal tract, including appendiceal tumors. A combination of PAX8 and SATB2 immunohistochemical stains can be used as a diagnostic tool to distinguish between these cases.

**Results:**

Immunostaining for SATB2, PAX8, CK7, CK20 and CDX2 was performed on 50 ovarian mucinous neoplasms (OMN) (39 cystadenomas, 4 borderline and 7 adenocarcinomas), 63 mucinous colorectal carcinoma (CRC), and 9 appendiceal mucinous neoplasms (AMN) [8 low grade appendiceal mucinous neoplasms (LAMN) and 1 adenocarcinoma]. PAX8 was positive in 32% of OMN and negative in all CRC and AMN cases. SATB2 was expressed in 2.0% of OMN, 77.8% of AMN, and 49.2% of CRC cases. CK7 was positive in 78.0% of OMN, 33.3% of AMN, and 9.5% of CRC cases. CK20 was expressed in 24.0% of OMN, 88.9% of OMN, and 87.3% of CRC cases. CDX2 was positive in 14.0% of OMN, 100% of AMN, and 90.5% of CRC cases. PAX8 can differentiate between OMN and AMN with high specificity but low sensitivity. CDX2 is the most sensitive marker for CRC and AMN, whereas SATB2 has better specificity.

## Introduction

Primary ovarian mucinous neoplasms (POMNs) are among the ovarian epithelial tumors for which diagnosis with certainty remains difficult. The major challenge is to exclude metastatic mucinous tumors (MMTs) from extragenital sites, especially appendiceal origin, given the fact there is overlap between both tumors in regard to the morphological features and immunohistochemical expression [1–3]. Many published studies have tried to determine the best panel of immunohistochemical stains to differentiate between them.

Among the most popular combinations are CK7, CK20, and CDX2. CK7 has been shown to be expressed in the majority of POMNs; however, CK7 is also expressed in MMTs, especially those of upper gastrointestinal origin with a lesser frequency for the lower gastrointestinal tumors (LGITs) [4–6]. In contrast, the diffuse positivity of CK20 and CDX2 would rather favor metastatic LGITs over POMN with certain limitations [5, 7]. That the immunohistochemical results in these borderline cases do not follow the above-mentioned pattern necessitates consideration of what might constitute a better panel.

PAX8 has been shown to be positive in the majority of Müllerian epithelial tumors [8]. However, it was negative in most GITs [9]. The percentage of POMNs expressing PAX8 is quite variable, ranging from 0% to 70% in previous studies [6, 8, 10–12].

SATB2 (special AT-rich sequence binding protein 2) is a recently described transcription factor which is both sensitive and specific to colorectal epithelium [13, 14]. SATB2 has been proven to be negative in POMNs with the exception of cases arising from teratoma, making it a superior marker in comparison to CK20 and CDX2 [15–17].

In this study, we sought to prove that the combination of PAX8 and SATB2 can be a sensitive and specific panel in discriminating POMNs from MMTs.

## Main text

### Materials and methods

#### Case selection and review

We retrieved 122 cases of primary tumors, consisting of 50 ovarian, 63 colonic, and 9 appendiceal mucinous neoplasms, from the archives of King Abdullah University Hospital, obtained during the period of 2005 to 2015. All cases were reviewed by two pathologists (NA and ME) and one representative section was chosen from each case. All the procedures performed were approved by the ethical committee of Jordan University of Science and Technology [Institutional Review Board (IRB) code number 40/2016, dated 17/03/2016] in accordance with the 1964 Declaration of Helsinki and its later amendments. Formal written informed consent was not required with a waiver by the IRB.

#### Immunohistochemistry

Four µm sections were produced from paraffin blocks using a Dako autostainer—Plus (Dako, Denmark). After the tissue was dewaxed, antigen retrieval was carried out in PT-link (Dako, Glostrup, Denmark), using a high pH buffer for 20 min. Endogenous peroxidases activity was blocked by incubation with 2.5% hydrogen peroxide for 10 min. Slides were then washed with phosphate buffer saline (PBS) twice and then primary antibodies were applied as follows: PAX8 (Biocare, United States; clone BC12, dilution 1:200) for 45 min at room temperature; SATB2 (Quartett, Germany; Cat No. 1-SP005-07, dilution 1:100) for 45 min at room temperature; CK7 (Dako, Denmark; Cat No. 15-619, prediluted) for 30 min at room temperature; CK20 (Dako, Denmark; Cat No. 15-803, prediluted) for 30 min at room temperature; and CDX2 (Dako, Denmark; Cat No. 15-777, prediluted) for 30 min at room temperature. Slides were then washed twice with PBS and signal detection was carried out using the Envision Dual Link System HRP Kit, K8000 (Dako, Glostrub, Denmark). Slides were then washed with distilled water and, finally, counterstained with Mayer’s Hematoxylin.

Stains were evaluated by two independent pathologists; the results were considered “positive” if > 5% of the tumor cells expressed the marker, and “negative” if < 5% of the tumor cells expressed the marker [16].

#### Statistical analysis

Analyses for dependent and independent variables were performed using SAS statistical software (version 9.2; SAS institute, Cary, NC, U.S.A.). Frequencies and percentages were used for categorical variables (immunostain result for each cancer). Sensitivity, specificity, and negative and positive predictive value measures of the primary ovarian mucinous neoplasms were computed using SAS functions and are shown in Tables 1 and 2. The 95% confidence interval was calculated for each screening method and is shown in Table 2. The 95% confidence interval was measured using the z-distribution $\left( {Ex:\;\left( {{\text{Sensitivity}}\; \pm \;Z\; * \;SE\;sensitivity} \right)} \right)$ The standard error for the screening method was calculated as

$$SE\;Sensitivity = \sqrt {\frac{{Sensitivity \left( {1 - Sensitivity} \right)}}{n}}$$

where n represents the denominator (people who have ovarian cancer in case of sensitivity).

**Table 1:** Immunophenotype of primary ovarian, colonic and appendiceal tumors

	Ovary	Colon	Appendix
CK7 n (%)			
Positive	39 (78.0)	6 (9.5)	3 (33.3)
Negative	11 (22.0)	57 (90.5)	6 (66.7)
CK20 n (%)			
Positive	12 (24.0)	55 (87.3)	8 (88.9)
Negative	38 (76.0)	8 (12.7)	1 (11.1)
CDX2 n (%)			
Positive	7 (14.0)	57 (90.5)	9 (100.0)
Negative	43 (86.0)	6 (9.5)	0 (0.0)
PAX8 n (%)			
Positive	16 (32.0)	0 (0.0)	0 (0.0)
Negative	34 (68.0)	63 (100.0)	9 (100.0)
SATB2 n (%)			
Positive	1 (2.0)	31 (49.2)	7 (77.8)
Negative	49 (98.0)	32 (50.8)	2 (22.2)
Total	50	63	9

**Table 2:** Sensitivity, specificity, positive predictive value (PPV), and negative predictive value (NPV) with their corresponding 95% confidence intervals for each individual immunohistochemical marker for detection of ovarian tumors

Immuno- histological marker	n (%)		% (95 Confidence interval: (Lower − Upper))			
	Ovary	Colon and appendix	Sensitivity	Specificity	PPV	NPV
CK7						
Positive	39 (32%)	9 (12.5%)	78 (66−89)	87.5 (80−95)	81.3 (70 −92)	85.1 (77−93)
Negative	11 (22%)	63 (87.5%)				
CK20						
Positive	12 (24%)	63 (87.5%)	24 (12−38)	12.5 (5−20)	16 (8−24)	19.1 (8−30)
Negative	38 (76%)	9 (12.5%)				
CDX2						
Positive	7 (14%)	66 (91.7%)	14 (4−23)	8.3 (2−15)	9.6 (3−16)	12.2 (3−21)
Negative	43 (86%)	6 (8.3%)				
PAX8						
Positive	16 (32%)	0 (0%)	32 (19−45)	100 (100 −100)	100 (100−100)	67.9 (59−77)
Negative	34 (68%)	72 (100%)				
SATB2						
Positive	1 (2%)	38 (52.8%)	2 (0−6)	47.2 (36−59)	2.6 (0−8)	41 (30−52)
Negative	49 (98%)	34 (47.2%)				

The sensitivity, specificity, PPV, and NPV was calculated for the primary mucinous ovarian neoplasms. Immunostains were considered “positive” if > 5% of the tumor cells expressed the marker, and “negative” if < 5% of the tumor cells expressed the marker

## Results

**Fig. 1:**
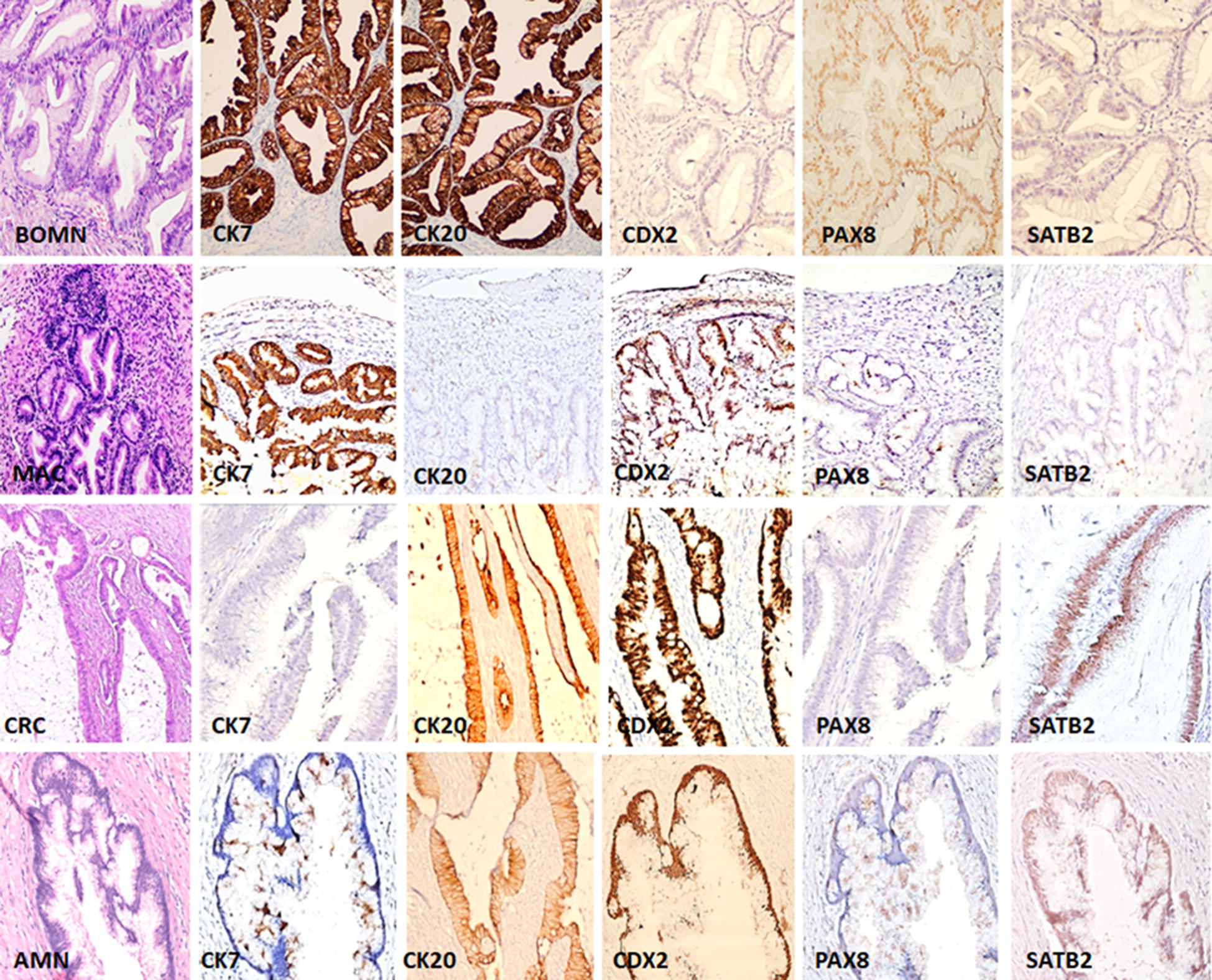
The pattern of expression of CK7, CK20, CDX2, PAX8 and SATB2 immunostains in borderline ovarian mucinous neoplasm (BOMN), ovarian mucinous adenocarcinoma (MAC), colorectal adenocarcinoma (CRC), and appendiceal mucinous neoplasm (AMN) (200 × magnification). Both BOMNs and MACs were positive for CK7+, but SATB2−, with variable expression of CK20. CDX2 was negative in all BOMNs while third of MAC were positive. PAX8 was negative in all MAC; with 2/4 BOMNs cases were positive. CRCs and AMNs showed CK7−, CK20+, CDX2+, PAX8−, and SATB2+. All images

Immunohistochemical expression data for ovarian, colonic, and appendiceal tumors are summarized in Tables 1 and 2. Representative images of the expression patterns are shown in Fig. 1.

### Ovarian tumors

We retrieved 50 primary ovarian mucinous tumors; 39 cases (78.0%) were mucinous cystadenomas, four (8.0%) were borderline tumors, and seven (14.0%) were mucinous cystadenocarcinoma. All patients were presented with ovarian mass. The clinicopathological features for patients with POMNs are summarized in Additional file 1: Table S1. CK7 was positive in the vast majority of POMNs (39 cases; 78.0%) while negative in 11 cases (22.0%), with 78.0% sensitivity and 87.5% specificity. CK20 was positive in 12 cases (24.0%) while negative in 38 cases (76.0%), with 24.0% sensitivity and 12.5% specificity. CDX2 was positive in seven cases (14.0%) while negative in 43 cases (86.0%), with 14.0% sensitivity and 8.3% specificity. SATB2 was positive in one case (2.0%) while negative in 49 cases (98.0%), with 2.0% sensitivity and 47.2% specificity. PAX8 was positive in 16 cases (32.0%) while negative in 34 cases (68.0%), with 32.0% sensitivity and 100.0% specificity.

All mucinous borderline tumors (4/4; 100%) were (CK7+, CDX2− and SATB2−). Two cases were (CK20+, PAX8−) and the other two were (CK20−, PAX8+).

All malignant mucinous cystadenocarcinomas (7/7; 100%) expressed CK7, and were negative for PAX8 and SATB2. Five out of seven cases (71.4%), were positive for CK20 and only two (2/7) were positive for CDX2 (28.6%).

Mucinous cystadenomas showed more heterogeneous results, in which 71.8% (28/39) and 35.9% (14/39) were expressing CK7 and PAX8, respectively, while 10.3% (4/39) were positive for CK20 and CDX2. SATB2 was negative in all cases. Two of the mucinous cystadenomas were associated with teratoma; both were consistently negative for CK20, CDX2, and PAX8. One case was positive for SATB2, and one was positive for CK7 while the other was negative.

### Appendiceal tumors

We retrieved nine cases of primary AMNs; eight cases were LAMN and one case was adenocarcinoma. The clinicopathologic characteristics for patients with AMNs are summarized in Additional file 1: Table S2. All nine cases (100%) were positive for CDX2 and negative for PAX8. CK7 was positive in three cases (33.3%) while negative in six cases (66.7%). CK20 was positive in eight cases (88.9%) while negative in one case (11.1%). SATB2 was positive in seven cases (77.8%) while negative in two cases (22.2%).

All LAMN cases exhibited a similar immunoprofile (CK20+, CDX2+, PAX8−), while the adenocarcinoma case co-expressed CDX2 and SATB2 with negativity for CK7, CK20 and PAX8.

### Colorectal tumors

We retrieved 63 cases of primary mucinous colonic adenocarcinoma. The clinicopathologic characteristics for patients with colonic mucinous adenocarcinoma are summarized in Additional file 1; Table S2. All 63 cases (100%) of CRC were negative for PAX8. CK20 was positive in 55 cases (87.3%) while negative in eight cases (12.7%). CK7 was positive in six cases (9.5%) while negative in 57 cases (90.5%). CDX2 was positive in 57 cases (90.5%) while negative in six cases (9.5%). SATB2 was positive in 31 cases (49.2%) while negative in 32 cases (50.8%).

## Discussion

Discriminating POMNs from MMTs is one of the problematic issues facing the pathologist in their practice and it is a crucial decision as respective management is totally different. Patients with POMNs have a better prognosis and usually do not require adjuvant chemotherapy [18–20], whereas patients with MMTs involving the ovary have a poor prognosis and may need systemic treatment.

The most popular panels used to discriminate POMNs from MMTs are CK7, CK20 and CDX2. CK7 was positive in 86% of POMNs, while 33% were positive for CK20 and CDX [6]. Vang et al. [4] showed that CK20 can be expressed in 83% of POMNs in both focal and diffuse patterns, while CDX2 was expressed in 40% of the cases. However, colonic carcinoma and appendiceal tumors are positive for CK7 in about 35% [21] and 31% of cases [12], respectively. CK20 is positive in colonic cancer in 66%–92% of cases [4]. Our study showed similar results: CK7 was the most sensitive and the second-most specific immunohistochemical marker for POMNs, with a sensitivity of 78.0% and a specificity of 87.5%. In contrast, CK7 expression in colonic and appendiceal tumors had 12.5% sensitivity and 22.0% specificity. CK20 was positive in 87.5% of colonic and appendiceal tumors and in 24.0% of POMNs.

CDX2 is typically expressed in a strong diffuse pattern in colorectal and appendiceal adenocarcinomas, with sensitivity of 100% [15], in contrast to their analogs in the upper GI and ovaries, where it is expressed focally. Vang et al. [7] showed that 40% of POMNs were positive for CDX2, while Strickland et al. [15] showed that 50% of ovarian mucinous adenocarcinomas were positive for CDX2. Furthermore, combined use of CDX2 with CK7 can help in this differential diagnosis, as 60% of the primary ovarian mucinous tumors are (CK7+, CDX2−, in contrast to mucinous adenocarcinomas of the lower gastrointestinal tract, where 83% of cases are (CK7−, CDX2+) [4, 7, 22]. In our study, CDX2 was positive in 14.0% of POMNs, and comparatively, in 88.0% of colonic and appendiceal tumors. CDX2 was the most sensitive marker (91.7%) for colonic and appendiceal tumors.

SATB2 is expressed by lower gastrointestinal normal epithelium, as well as by primary and metastatic CRC [13, 14]. In CRC, sensitivity for SATB2 is analogous with CK20 and CDX2, for which rates of positivity range from 85%–93% [13, 14, 23]. Staining for SATB2 is useful in ovarian tumors with morphologic evidence of intestinal differentiation, or with expression of CDX2 or CK20, as most cases (98%) are typically negative [24]. In our study, SATB2 was positive in 2.0% of POMNs compared to in 52.8% of colonic and appendiceal tumors. Appendiceal tumors showed more positivity for SATB2 (77.8%) in comparison to colonic tumors (49.2%), making it a useful maker in discriminating POMNs from appendiceal and colonic tumors. The percentage of positivity in our study was lower than in the literature and this might be due to the use of a different antibody.

PAX8 is a sensitive and specific marker for tumors of Müllerian origin [8] but it is less commonly expressed in ovarian mucinous tumors, with a range therein from 0% to 70% [6, 8, 10–12]. This discrepancy in the results is most likely due to the use of anti-PAX8 rabbit polyclonal antibody instead of using the monoclonal anti-PAX8 antibody (mAb) [10, 11]. Recent studies by Hu et al. [11] and Strickland et al. [12] showed that PAX8 (mAb) is expressed in 53% and 70% of POMNs, respectively. Yet, Chu et al. [22] found that PAX8 positivity was mainly seen in mucinous tumors from the female genital tract. That said, PAX8 is uniformly negative in GITs as shown by Peiguo et al. [22]. Similarly, in our study, all colonic and appendiceal tumors were negative for PAX8. However, PAX8 was positive in 32.0% of POMNs in our study, which is lower than that reported by Hu et al. [11] and Strickland et al. [12].

In conclusion, the most sensitive marker for POMNS is CK7 (78.0%), whereas the most specific is PAX8 (100%). Regarding mucinous tumors arising from the colon and appendix, the most sensitive marker is CDX2 (91.7%), while the most specific is SATB2 (98.0%). Therefore, adding PAX8 and SATB2 to the panel of immunostains for differentiating mucinous tumors will be of great benefit. If the tumor is positive for PAX8, it is most likely to be of ovarian origin. On the other hand, positivity for SATB2 is most likely of colonic/appendiceal tumors.

## Limitation

In the present study, we had a relatively small sample size, especially for AMNs and ovarian mucinous adenocarcinoma cases. We recommend the use of larger sample sizes in future research involving multiple institutions, instead of a single institution as in our study.

## Supplementary information

**Additional file 1: Table S1.** Clinicopathologic characteristics of the ovarian mucinous neoplasms. **Table S2**. Clinicopathologic characteristics of colorectal and appendiceal mucinous tumors.

## Data Availability

Data is available from the corresponding author upon reasonable request.

## Abbreviations

OMN: ovarian mucinous neoplasms
CRC: colorectal carcinoma
AMN: appendiceal mucinous neoplasms
LAMN: low grade appendiceal mucinous neoplasm
POMN: primary ovarian mucinous neoplasm
MMT: metastatic mucinous tumors
LGIT: lower gastrointestinal tumor
BOMN: borderline ovarian mucinous neoplasm
MAC: ovarian mucinous adenocarcinoma
