# Supplementary material for: The utility of PAX8 and SATB2 immunohistochemical stains in distinguishing ovarian mucinous neoplasms from colonic and appendiceal mucinous neoplasm
Source: BMC Res Notes. 2019 Nov 26;12:770. doi: 10.1186/s13104-019-4816-9 (PMC6880435; doi:10.1186/s13104-019-4816-9)
Supplement: Supplementary file 1 — Additional file 1: Table S1. Clinicopathologic characteristics of the ovarian mucinous neoplasms. Table S2. Clinicopathologic characteristics of colorectal and appendiceal mucinous tumors. [file 13104_2019_4816_MOESM1_ESM.docx]

**Table S1:** Clinicopathologic characteristics of the ovarian mucinous neoplasms

| **Variables** | **BENIGN n(%)** | **BORDERLINE n(%)** | **MALIGNANT n(%)** |
| --- | --- | --- | --- |
| **AGE (YEAR)**  Range  Mean | 10-77  40.6 | 21-61  38 | 30-60  37.7 |
| **SIZE (CM)**  <10  ≥10  Mean  Range | 25 (64.1%)  14 (35.9%)  10  5-28 | 1 (25%)  3 (75%)  14.1  8.5-20 | 0 (0%)  7 (100%)  24.87  11-45 |
| **LATERALITY**  Unilateral  Bilateral | 37(94.9%)  2 (5.1%) | 4 (100%)  0 (0%) | 6 (85.7%)  1 (14.3%) |
| **APPENDECTOMY**  Yes  No | 3 (7.7%)  36 (92.3%) | 1 (25%)  3 (75%) | 4 (57.1%)  3 (42.9%) |
| **TUMOR** **GRADE**  I  II  III | Not applicable | Not applicable | 3 (42.8%)  3 (42.8%)  1 (14.4%) |
| **TUMOR STAGE**  T1a  T1c  T3c | Not applicable | 3 (75%)  1 (25%) | 4 (57.1%)  2 (28.5%)  1 (14.4%) |
| **TOTAL : 50** | 39 (78) | 4 (8) | 7 (14) |

**Table S2:** Clinicopathologic characteristics of colorectal and appendiceal mucinous tumors.

| **Variables** | **Colorectal mucinous adenocarcinoma n(%)** | **Low grade appendiceal mucinous neoplasms n(%)** | **Appendiceal mucinous adenocarcinoma n(%)** |
| --- | --- | --- | --- |
| **AGE (YEAR)**  Range  Mean | 29-97  60 | 23-75  51 | NA  49 |
| **TUMOR** **GRADE**  Well  Moderate  Poor | 10 (15.8)  45 (71.4)  8 (12.8) | NA | 0 (0)  1 (100)  0 (0) |
| **TUMOR STAGE**  pTis  pT1  pT2  pT3  pT4 | 0 (0)  1(1.6)  2 (3.2)  38 (60.3)  22 (34.9) | 5 (62.5)  NA  NA  1 (12.5)  2 (25) | 0 (0)  0 (0)  0 (0)  0 (0)  1 (100) |
| **LYMPH NODE METASTASIS** | 38 (60.3) | NA | 0 (0) |
| **DISTANT METASTASIS** | 6 (9.5) | NA | 0 (0) |
| **TOTAL :** | 63 | 8 | 1 |

NA, not applicable
